# The Effect of Disease and Injury on Faecal Cortisol Metabolites, as an Indicator of Stress in Wild Hospitalised Koalas, Endangered Australian Marsupials

**DOI:** 10.3390/vetsci10010065

**Published:** 2023-01-16

**Authors:** Flavia Santamaria, Rolf Schlagloth, Ludovica Valenza, Rupert Palme, Deidre de Villiers, Joerg Henning

**Affiliations:** 1 Koala Research-Central Queensland and Flora, Fauna and Freshwater Research Cluster, School of Health, Medical and Applied Sciences, Central Queensland University, North Rockhampton, QLD 4702, Australia; 2 Australia Zoo Wildlife Hospital, Beerwah, QLD 4519, Australia; 3 Department of Biomedical Sciences, University of Veterinary Medicine, 1210 Vienna, Austria; 4 Endeavour Veterinary Ecology, Toorbul, QLD 4510, Australia; 5 School of Veterinary Science, The University of Queensland, Gatton, QLD 4343, Australia

**Keywords:** koala, faecal cortisol metabolites, enzyme immunoassay, rehabilitation, wildlife hospital, *Chlamydia*, injury, disease, stress, non-invasive method

## Abstract

**Simple Summary:**

Habitat loss, urbanisation and climate change may cause stress in koalas. Non-invasive monitoring of faecal cortisol metabolites (FCMs) can be utilised to evaluate the impact of stress. The effectiveness of two enzyme immunoassays (EIAs), 50c and cortisol, in measuring FCM values in wild, stressed koalas was tested. Faecal samples of 234 diseased, injured and control koalas in Queensland, Australia were analysed. Diseased and injured koalas had significantly higher FCM values than clinically healthy control animals as measured by the 50c EIA. Only the 50c EIA detected higher absolute values in males, and also found that females showed a more elevated response to stress manifested by injury and disease. The cortisol EIA was also found unreliable in detecting stress in rehabilitated koalas treated with synthetic glucocorticoids as it cross-reacts with these chemicals.

**Abstract:**

Loss of habitat, urbanisation, climate change and its consequences are anthropogenic pressures that may cause stress in koalas. Non-invasive monitoring of faecal cortisol metabolites (FCMs) can be utilised to evaluate the impact of stressors. The aim was to determine if the tetrahydrocorticosterone (50c) and cortisol enzyme immunoassays (EIAs) could be effective in measuring FCM values in wild, stressed koalas. This research included 146 koalas from the Australia Zoo Wildlife Hospital (AZWH) and 88 from a study conducted by Endeavour Veterinary Ecology (EVE), Queensland, Australia. Faecal samples of diseased, injured and control koalas were analysed. The effect of hospitalisation on FCM values was also investigated. Diseased and injured koalas had significantly higher FCM values than clinically healthy control animals as measured by the 50c EIA. FCM values with the cortisol EIA differed significantly between control and diseased koalas, but not between control and injured ones. Moreover, only the 50c EIA detected higher absolute values in males compared to females, and also found that females showed a more elevated response to stress manifested by injury and disease compared to males. The 50c EIA detected stress during hospitalisation better than the cortisol EIA. The cortisol EIA was also found unreliable in detecting stress in rehabilitated koalas treated with synthetic glucocorticoids as it cross-reacts with these steroids providing artificially high values.

## 1. Introduction

The hypothalamic-pituitary-adrenal (HPA) axis is one of the systems responding to an organism’s internal and external changes to maintain homeostasis. It regulates metabolic and physiological processes by stimulating the release of glucocorticoids (GCs), such as cortisol and corticosterone, produced by the adrenal cortex [1,2]. During acute stress events, the adrenocorticotropic hormone (ACTH), produced by the pituitary gland, stimulates the increased release of GCs from the adrenal gland cortex into the circulatory system. Cortisol is the main stress hormone in most mammals [3,4,5], it is metabolised by the liver and bacterial enzymes in the intestine [3], hence free cortisol is not excreted via the faeces [6]. 

During stressful situations, its function is to maintain homeostasis and ensure the individual’s survival by increasing the output of glucose for muscular and brain function and decreasing the activity of peripheral organs, hence preparing the organism’s response to cope with the situation. Anti-inflammatory responses and immune suppression are also exacerbated by cortisol release in response to stress [7,8,9].

The secretion of cortisol is naturally limited by a negative feedback loop caused by its own secretion [5], and designed to limit the immunosuppressive activity of this hormone [8]. Chronic stress occurs when stressors persist, and cortisol secretion continues. The increased, constant output of cortisol consequential to physiological, physical and/or environmental chronic stress elicits a dysfunctional HPA response. Studies on young animals chronically exposed to stressors have also shown a reduced output of GCs in response to stressful situations as adults [10]. The implication for wildlife exposed to increasing anthropogenic stressors is that the output of GCs may be compromised [11] and, more importantly, that it has a negative effect on their health [8]. Anthropogenic stressors have a direct impact on the health of wildlife with the increase in common and novel disease outbreaks causing the decline of many populations [12].

In February 2022, koalas (*Phascolarctos cinereus*) were listed as endangered in Queensland, New South Wales (NSW) and the Australian Capital Territory under the Environment Protection and Biodiversity Conservation Act 1999 (EPBC Act) [13]. The continuous decline in koala numbers is directly linked to anthropogenic actions related to loss or fragmentation of habitat and growing urbanisation [14].

In South East Queensland (SEQ), in particular, there is significant loss of koala habitat for housing developments and for the expansion of the road and rail networks. The increased traffic volume and larger distances between areas of koala habitat are causing koalas to travel long distances and to cross roads, with a high chance of being attacked by dogs or hit by vehicles [15,16,17,18,19]. These human-driven pressures are further compounded by anthropogenic climate change manifesting itself through high temperatures, more frequent and prolonged droughts and floods and catastrophic bushfires. The aforementioned events are stressors for koalas and can lead to injuries, diseases and death [16,18,20]. *Chlamydia pecorum* is one of the bacteria causing devastating diseases in koalas, affecting the urogenital system with cystitis, endometritis, pyelonephritis and prostatitis, as well as causing blindness and impacting the respiratory tract [21,22]. 

Conversely, there are ample examples showing that the effect of pain caused by illness and injuries in animals can increase stress hormone and, as a consequence, faecal cortisol metabolite (FCM) concentration [23,24,25,26,27]. 

The effect of stressors on koalas, at an individual and population level, can be evaluated using a non-invasive method that measures the levels of FCMs. Accounting for the lag time due to the transition in the intestinal tract, metabolites of cortisol are directly related to adrenocortical activity during stressful events [20,27,28].

Previous studies, which explored the relationship between stress (investigated by FCMs) and injuries or illness in domestic species and wildlife, including koalas, yielded mixed results with metabolite values increasing above or decreasing below the baseline [25,26,29,30,31]. 

Our recent research, conducted with captive koalas in wildlife parks, identified tetrahydrocortisol to be the main FCM in koalas [32]. Validation of several enzyme immunoassays (EIAs) established a tetrahydrocorticosterone EIA (aka 50c) to be the most suitable in evaluating FCM levels in koalas [32]. The suitability of a cortisol EIA and the 50c EIA in detecting FCM values was further investigated, and the latter was again found to be better suited [33,34].

However, those studies did not investigate the suitability of both EIAs in identifying naturally occurring stressors in this species. A clear need was expressed for more studies to validate the EIA using biological parameters [35] such as diseases and injuries. Therefore, here, both the 50c and the cortisol EIA were tested with the aim to determine the effect of disease and injury as well as the effect of hospitalisation on adrenocortical activity as an indicator of stress in wild koalas in SEQ.

## 2. Materials and Methods

### 2.1. Koalas

The study started in February 2021 and was completed in March 2022. Two groups of wild koalas were part of this study: a group of 146 koalas admitted to the Australia Zoo Wildlife Hospital (AZWH) (code: AZ), Beerwah, Queensland, and one group of 88 from a study conducted by Endeavour Veterinary Ecology (EVE) at The Mill (code: ML), a locality at Moreton Bay, Queensland. Details of the makeup of these groups are shown in Table 1. The breeding season for koalas in the study region commences in September and ends in January [34]. The names of the koalas in this study were assigned by AZWH and EVE.

#### 2.1.1. AZ Koalas

The 36 koalas that were neither diseased nor injured and taken to AZ by concerned citizens were considered as the AZ control. During the rehabilitation, ill and injured koalas were treated with a variety of systemic and local (ocular) medications. Systemic treatment included oral synthetic GC, prednisolone (Redipred, NewChem SpA, Verona, Italy), antimicrobial sub-cutaneous injections of doxycycline (Vetafarm, Wagga Wagga, NSW Australia) and chloramphenicol (Ceva Animal Health Pty Ltd., Glenorie, NSW, Australia) and enrofloxacin antibiotic (Baytril, Bayer, Leverkusen, Germany) used as nebulizer. Local treatment included eye ointments with chloramphenicol and GC (Chloroptsone) (Ceva Animal Health Pty Ltd., Glenorie, NSW, Australia) and antibiotic chloramphenicol (Chlorsig, Sigma Pharmaceuticals Pty Ltd., Clayton, VIC, Australia). Wherever possible, information was obtained on clinical activities performed during the collection of scats for the longitudinal study. Chlamydial infection was determined using loop-mediated isothermal amplification (LAMP) [36] with Genie II (OptiGene, Horsham, South of England, UK). Values were provided in time (min) to amplification.

In total, 346 faecal samples were obtained for this study from the AZ koalas. Only intact fresh pellets were collected on admission and in the morning from the ground of the enclosure where the koalas were housed individually. Despite the likely need for an increased collection effort, the use of fresh pellets is recommended to avoid the possible effect of environmental conditions on the structure of the samples [34].

A first sample from each koala was collected on arrival at the hospital before any intervention was undertaken by the veterinarians. Due to the lag time that occurs between a stressful event and the increase in FCM values [32], the analysis of these samples reflects stressful incidents occurring many hours before admission.

A second sample from 53 of the 146 koalas was collected again between 10 and 15 days after admission to detect if changes in FCM values occurred during hospitalisation (control: N = 7, diseased: N = 33, injured: N = 13). Serial faecal pellets from 20 of the 146 admitted koalas (12 diseased, 6 injured and 2 control) were also collected for a period between 7 and 10 days from admission (longitudinal study).

#### 2.1.2. ML Koalas

The 88 koalas from ML were part of The Mill Koala Tagging and Monitoring Program, carried out by EVE on behalf of Moreton Bay Regional Council. The program aimed to ensure the welfare of koalas during vegetation clearing operations for site remediation and construction works as the site transitioned from industrial use to a mixed-use precinct with a university campus, community infrastructure and future commercial development precincts. Koalas were fitted with collar-mounted K-Tracker biotelemetry tags weighing 70 g (LX Group, Sydney, NSW, Australia), a very high frequency (VHF) transmitter (Series A2600, Advanced Telemetry Systems, Australia) and lead weight in housing for a total collar weight of 190 g, with a customised weak link based on the weight of the animal. When a koala was located, it was caught by a tree climber using ropes and harness. Koalas were immediately placed in a transport cage (dimensions: 520 mm H × 580 mm L × 350 mm W) with fresh eucalypt browse and transported to the EVE facility for a veterinary examination.

Koalas remained in the transport cage until the veterinary examination was conducted. While undergoing veterinary assessment, any fresh and intact faecal pellets voided were collected. If pellets were not produced during the 20 to 45 min veterinary examination, the cage was checked to determine if any suitable fresh pellets could be collected.

A sample of at least four intact faecal pellets was collected if fresh pellets were observed. Data relating to each individual’s history and chlamydial status were provided to this project. None of these koalas were either injured or diseased at the time of the faeces collection, hence this was considered as an external control.

All the samples provided for this study were immediately placed in a −20 °C freezer, and remained at this temperature until the samples were collected and transported with a −20 °C portable freezer to a −80 °C freezer before processing.

### 2.2. Location of Koalas

Latitudinal and longitudinal coordinates for the 146 koalas prior to admission at AZ were derived from the precise locations provided by the rescuers. The coordinates of the ML koalas were provided by the EVE researchers. 

**Figure 1:**
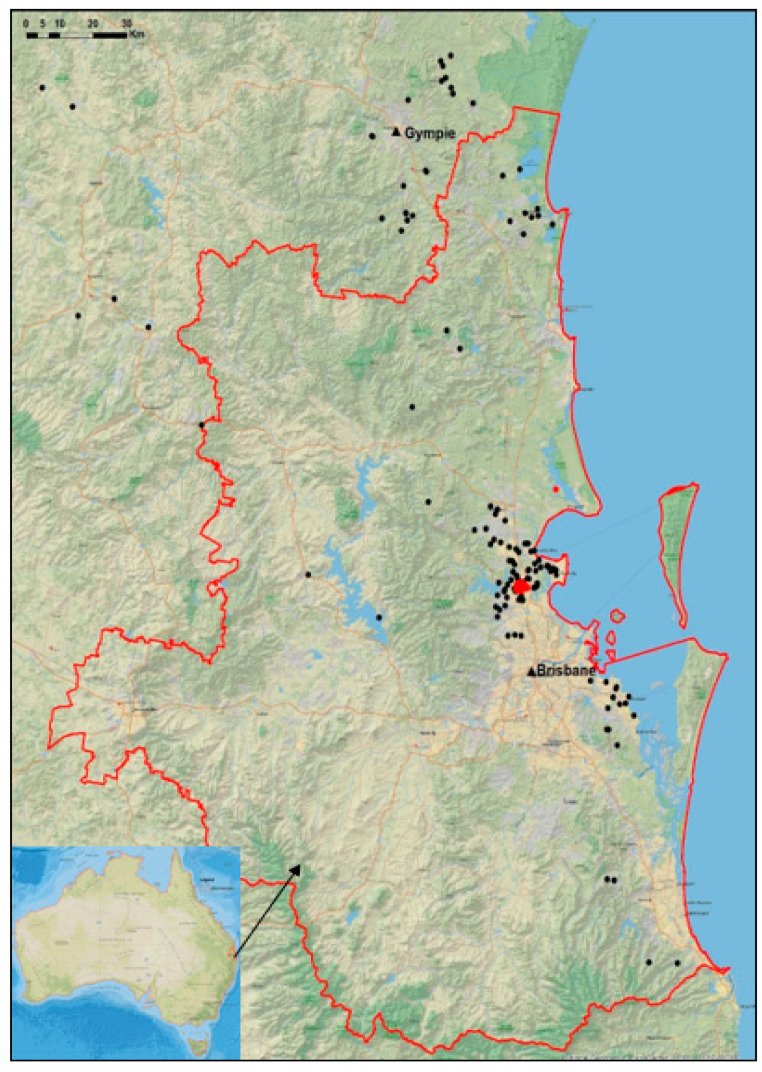
Map of South East Queensland (SEQ; red line) showing the provenance of the koalas admitted to the Australia Zoo Wildlife Hospital (black circles), and the location of the 88 The Mill koalas (red circles). The inserted map shows the location of South East Queensland in Queensland, Australia.

A map of the locations of all koalas involved in the study (Figure 1) was generated in ArcGIS (ArcMap 10.8.1; ESRI, Redlands, CA, USA).

### 2.3. FCM Extraction and Analysis

The extraction procedure and analysis are described in Santamaria et al. [33]. In brief, 500 mg of wet faeces were placed into a 10 mL tube and 5 mL of 80% methanol were added. Samples were shaken for 30 min with an orbital rotator shaker, vortexed for 2 min with a hand vortex and centrifuged at 2500× *g* for 15 min. Completely dried-down aliquots (0.25 mL) of the extracts in 1 mL Eppendorf tubes, sealed with paraffin film, were shipped to the University of Veterinary Medicine (Austria), where dried sample extracts were resolubilised in 80% methanol and further diluted with assay buffer (1 + 9). Aliquots were analysed in duplicate with cortisol (polyclonal antibody against cortisol-3-CMO raised in rabbits) and tetrahydrocorticosterone (50c) enzyme immunoassays (EIAs). The EIAs were selected based on the findings of Santamaria et al. [33] and used to evaluate FCM values in diseased and injured animals. Intra- and inter-assay coefficients of variation (CVs) were below 10% and 15%, respectively, for a high and low concentration pool sample in both assays. FCM concentrations are expressed as ng/g wet faeces. Details of the EIAs, including cross-reactivities have been described previously [27,37].

### 2.4. Statistics

**Figure A1:**
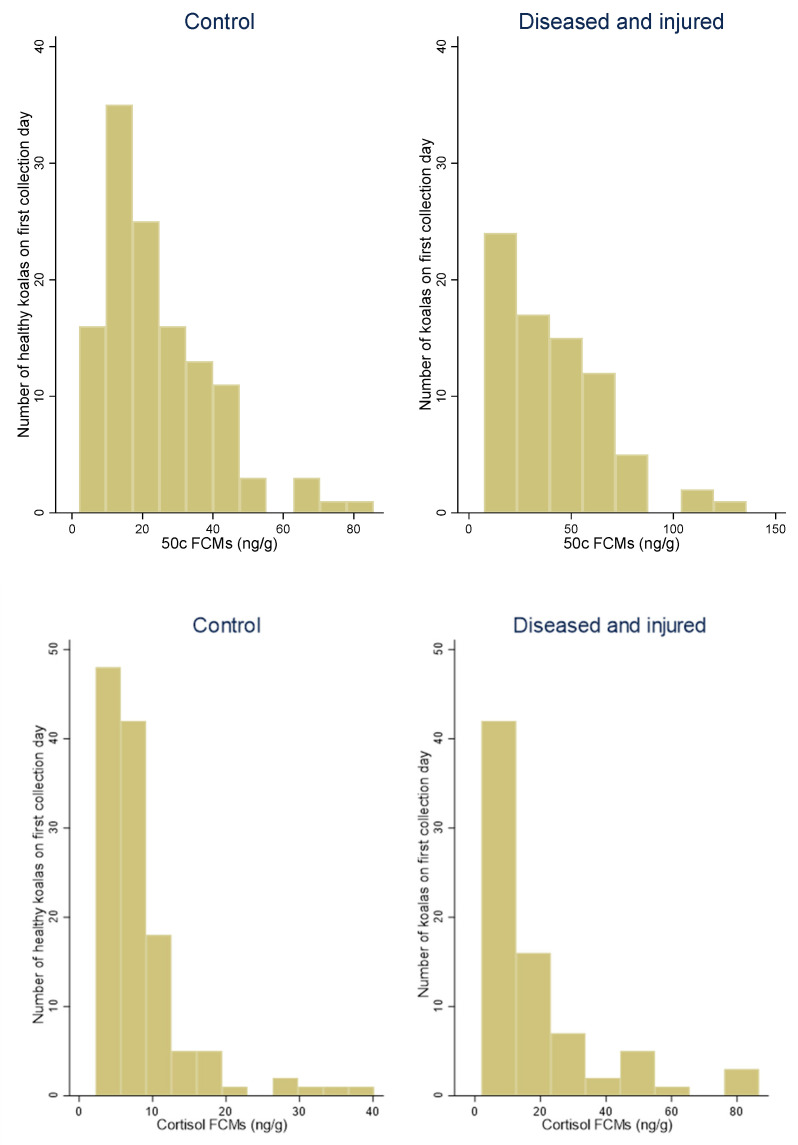
The FCM (faecal cortisol metabolites) values of the control only and of diseased and injured koalas, measured with 50c EIA and cortisol EIA (enzyme immunoassays). FCM values were not normally distributed (Shapiro–Wilk Test; *p*-values < 0.001).

The normality of FCM values was assessed by visually examining the histograms (Appendix B, Figure A1) and by using the Shapiro–Wilk test.

Descriptive statistics of FCM values were presented for all subgroups of animals, including 95% confidence intervals for medians [38].

For control animals, the non-parametric Mann–Whitney U (Wilcoxon rank sum) test was used to compare FCM values between the locations (AZ, ML) and between breeding and non-breeding season for samples obtained on the first collection day.

The non-parametric Kruskal–Wallis test was applied to compare FCM values between koala’s conditions (control, diseased, injured) for samples obtained on the first collection day. Nonparametric pairwise comparisons were then conducted using Dunn’s test with the Bonferroni adjustment [39]. The Mann–Whitney U test was then used to compare, separately for each condition, FCM between males and females for samples obtained on the first collection day.

Poisson regression was utilised in a multivariate model to evaluate the association between FCM values and koala condition, sex and the interaction between sex and condition for samples obtained on the first collection day [40]. Robust standard errors were used in the Poisson regression and results were displayed as incidence rate ratios (IRR). The Wald test was applied to evaluate the significance of categorical variables in the multivariate model.

The Mann–Whitney U test for matched data (Wilcoxon rank sign) was applied to compare EIA values for AZ koalas between the first sampling and the subsequent sampling 10–15 days later.

The correlation between 50c and cortisol was explored in a scatter plot and the Pearson correlation coefficient was used for quantification.

Microsoft Excel (Version 2208 Build 16.0.15601.20204) was used for descriptive statistics and bar and line graphs. Data analysis was performed in STATA 16.1 (StataCorp LLC, 4905 Lakeview Drive, College Station, TX 77845, USA).

## 3. Results

A total of 234 koalas from the AZ and ML were considered for the analysis, of which 36 from AZ and 88 from ML were neither diseased nor injured and were considered controls. Of the AZ koalas, 76 were diseased and 30 were injured. Four were diseased and injured, but were excluded from further analyses.

### 3.1. FCM Values for the Control Koalas from AZ and ML

**Figure 2:**
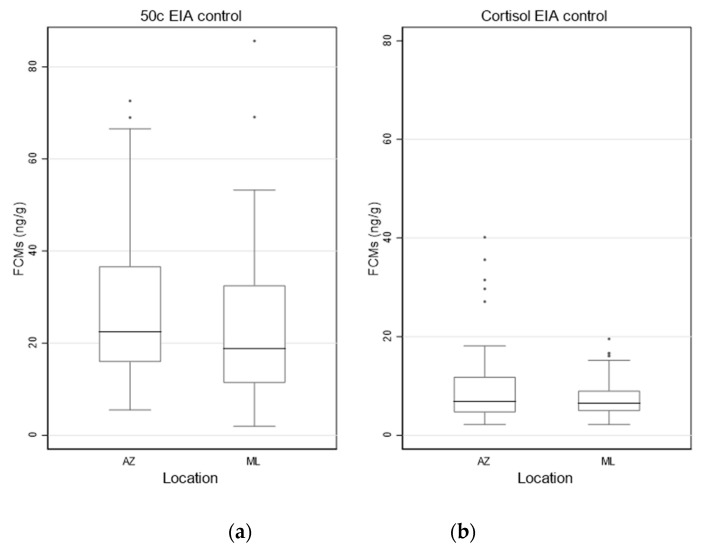
Box and whisker plots showing FCM (faecal cortisol metabolite) values (ng/g) of AZ (N = 36) and ML control koalas (N = 88) measured with the 50c (**a**) and cortisol (**b**) EIAs (enzyme immunoassays) on the first collection day.

The distribution of FCM concentrations measured with both EIAs for the AZ and ML control are shown in Figure 2a,b.

The median values (ng/g) (95% CI) detected by the 50c EIA and cortisol EIA in koalas at AZ (N = 36) on the first collection day were 22.4 (17.3, 29.3) and 6.8 (5.0, 9.3), respectively. The median values (ng/g) (95% CI) in koalas at ML (N = 88) on the first collection day were 18.8 (15.6, 23.2) (50c EIA) and 6.4 (5.6, 7.1) (cortisol EIA), respectively. Mean, minimum, maximum FCM values for control koalas from AZ and ML are presented in Appendix A, Table A1.

There was no significant difference in FCM values measured by the 50c (*p* = 0.1379) and the cortisol (*p* = 0.5858) EIAs between the AZ and the ML control, highlighting that healthy koalas have similar FCM values in different geographical locations and confirming that both could be used as a combined control in the analysis.

The breeding season for koalas in the study region commences in September and ends in January [34]. FCM values measured with 50c EIA and cortisol EIA in control koalas did not differ significantly (*p* = 0.1469 and *p* = 0.5415) between the breeding and non-breeding season (Appendix B, Table A6).

### 3.2. Longitudinal and 2-Day Scat Collection with and without GC Treatment

**Figure 3:**
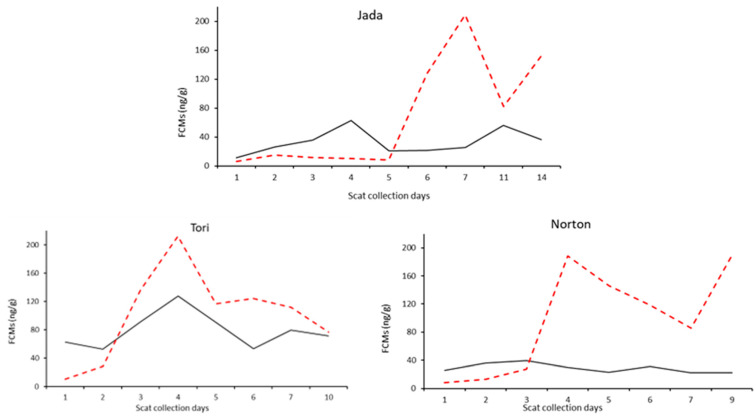
FCM (faecal cortisol metabolite) values of three koalas during the longitudinal study, showing an artificial increase one day after the systemic administration of synthetic GCs measured with the cortisol EIA (enzyme immunoassay; red dash line). The values of FCMs measured with the 50c EIA (black solid line) are within the normal variation expected for stressed koalas.

**Figure 4:**
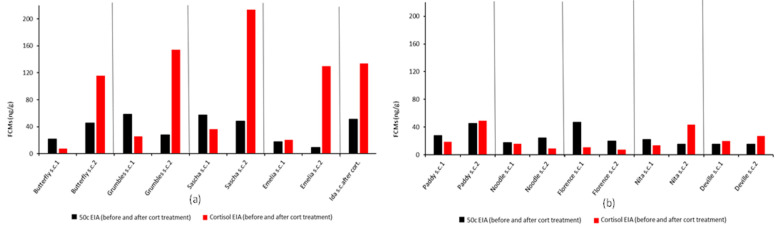
FCM (faecal cortisol metabolite) values of the 2-day scat (faeces) collection (s.c.1 and s.c.2) of 10 koalas (names shown on the x axis) measured by 50c (black bars) and cortisol EIAs (enzyme immunoassays) (red bars). Second samples (s.c.2) shown in (**a**) were collected during the systemic administration of GC and show artificial increase in FCM values measured by the cortisol EIA. Second samples in (**b**) were collected after the systemic GC administration was terminated and were also measured by the cortisol EIA, but show no artificial increase in values. No artificial increases are seen with the 50c EIA following GC administration.

Thirty-five koalas were treated with systemic antibiotics, and, of these, 13 were also treated with systemic, synthetic GCs at different stages of the rehabilitation at AZ. The FCM values, measured by the cortisol EIA, of eight koalas treated with GCs until or after final scat collection, showed a large artificial increase (Figure 3 and Figure 4a). This artificial increase occurred the day after the administration of systemic GCs, and it is especially noticeable for three koalas during the longitudinal study (Figure 3). The relatively high values measured with this EIA on day 4 for Jada and Tori are likely related to general anaesthetic (GA) on day 2 and the injection also on day 2, respectively (Figure 3).

The outcome of the longitudinal study of 17 hospitalised koalas that were not treated with GCs is shown in Appendix A (Table A2). Most koalas reacted to treatments and interventions with a spike in FCM values measured with 50c EIA the day after the event. Correlation coefficients are also shown in Appendix A, Table A2. Despite the significant correlation between the two EIAs (displayed below), in many cases, the cortisol EIA did not detect a change in FCM values after treatments or interventions during rehabilitation. The highest FCM value obtained was from a diseased koala (Cloyna), measured with the 50c EIA on day 2 of rehabilitation, was 373 ng/g (min = 113; max = 373: median = 208), but only 4.31 ng/g (min = 1.87; max = 51.4: median = 7.9) measured by the cortisol EIA. Values were high during the whole period of hospitalisation until he was euthanised after 12 days from admission (Appendix A, Table A2).

The artificial increase is also evident in the 2-day scat collection of five koalas (Figure 4a). FCM values measured with the 50c EIA did not show any increase attributed to prednisolone treatment. Koala Ida’s first sample was lost. However, hospital records show that she received GCs from the day of admission and was on the medication at the time the second sample was collected.

The FCM values of the five koalas whose treatment ended before the last scat collection (Appendix A, Table A3) did not show this artificial increase measured by the cortisol EIA (Figure 4b).

The FCM values for samples collected on arrival at the AZ hospital and between the 10th and 15th day from the time of hospitalisation of all the koalas that were not treated with systemic GCs (N = 52) are shown in Table 2. There was no significant difference at *p* < 0.05 between the first and second sampling for any of the conditions for 50c EIA and for control and diseased animals measured with the cortisol EIA.

### 3.3. Correlation between FCM Values Analysed with the 50c and Cortisol EIAs in Day 1 Samples of Control, Diseased and Injured Conditions

**Figure 5:**
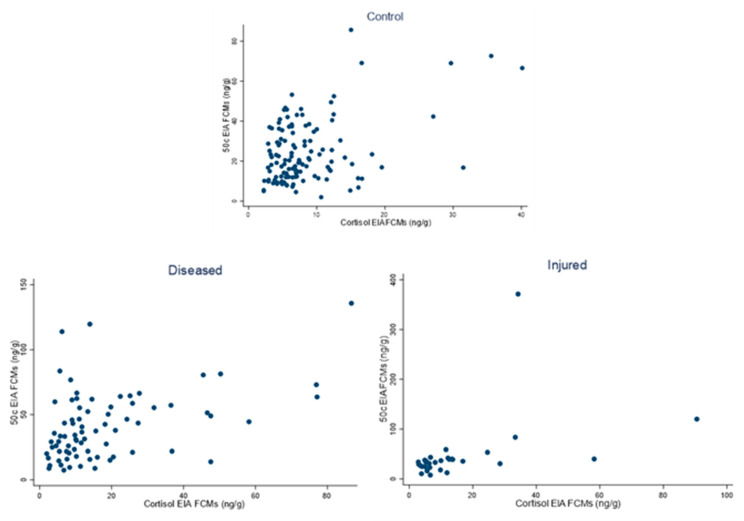
Scatterplots correlating FCM (faecal cortisol metabolite) values of the control (N = 124), diseased (N = 76) and injured (N = 30) koalas, measured with the 50c and cortisol EIAs (enzyme immunoassays).

Scatterplots were used to display the bivariate correlations between the two EIAs for the control (N = 124), diseased (N = 76) and injured (N = 30) in day 1 samples (Figure 5). The data were restricted to day 1 samples as the effect of treatment in the hospital did not appear to have impacted EIA values.

The correlation coefficient for the control was 0.4120 (*p* < 0.001), for the diseased was 0.4479 (*p* < 0.001) and for the injured was 0.4310 (*p* < 0.0174).

### 3.4. Impact of Disease and Injuries on FCM Values

**Figure 6:**
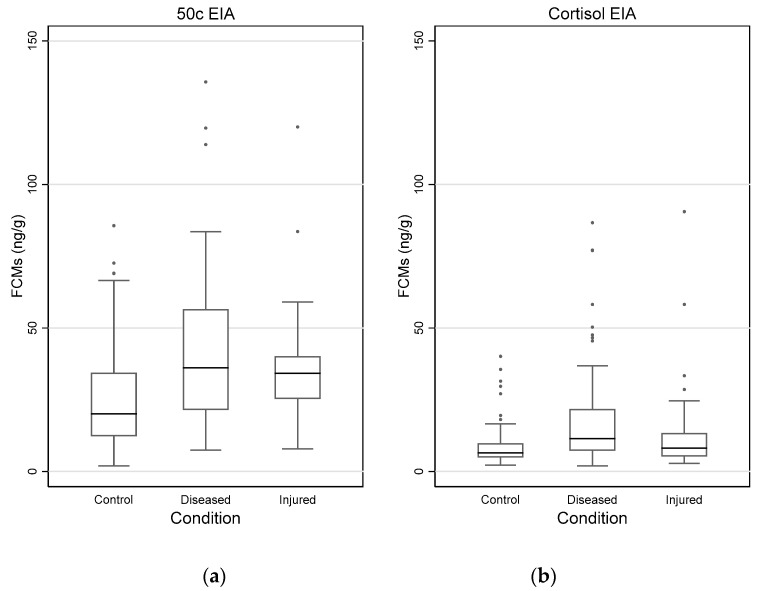
Box and whisker plots showing FCM (faecal cortisol metabolite) values (ng/g) of control (N = 124), diseased (N = 76) and injured (N = 30) koalas, measured with the 50c (**a**) and cortisol (**b**) EIAs (enzyme immunoassays) on the first collection day. Note: Megan’s value of >200 ng/g was excluded to avoid distortion of the values in the box and whisker plot.

The distribution of FCM values measured with 50c and cortisol EIAs for control, diseased and injured koalas on the first collection day is shown in Figure 6a,b. The highest FCM value measured with the 50c EIA was 371 ng/g (34.0 ng/g measured by cortisol EIA), obtained on first day collection from koala Megan, injured by a car and euthanised on arrival to the AZ.

The median FCM values (ng/g) (95% CI) detected with the 50c EIA and cortisol EIA for the combined control koalas from AZ and ML (N = 124) were 20.0 (17.2, 23.3) and 6.5 (5.9, 7.1), respectively. The median FCM values (ng/g) (95% CI) detected with the 50c EIA for the diseased koalas from AZ (N = 76) were 36.1 (29.0, 44.5) and 11.4 (9.8, 14.5) with cortisol EIA. The median FCM values (ng/g) (95% CI) of the injured koalas (N = 30) as measured with the 50c EIA were 34.9 (29.6, 39.5) and with the cortisol EIA 8.9 (5.6, 12.5).

Mean, minimum and maximum FCM values for these three groups of koalas with different conditions are presented in Appendix A, Table A4.

FCM values measured by both EIAs differed significantly between control, diseased and injured koalas (Kruskal–Wallis, *p* < 0.001). In the pairwise comparison, diseased and injured koalas had significantly higher FCM values measured by the 50c EIA compared to the control (*p* < 0.0001 and 0.0007, respectively), but FCM values did not differ between diseased and injured koalas (*p* = 1.000). FCM values measured by the cortisol EIA only differed significantly between control and diseased koalas (*p* < 0.0001), but not between control and injured (*p* = 0.1016) or between diseased and injured koalas (*p* = 0.1246).

### 3.5. Impact of Disease and Injuries on FCM Values by Sex

**Figure 7:**
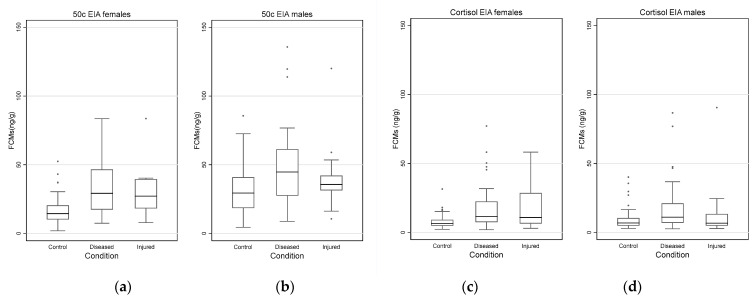
Box and whisker plots showing FCM (faecal cortisol metabolite) values (ng/g) for females and males in control (females N = 63; males N = 61), diseased (females N = 42; males N = 34) and injured (females N = 11; males N = 19) koalas, as measured with the 50c (**a**,**b**) and cortisol (**c**,**d**) EIAs (enzyme immunoassays) on the first collection day.

The distribution of FCM values measured with 50c and cortisol EIAs for control, diseased and injured female and male koalas on the first collection day is shown in Figure 7a–d.

Median FCM values (ng/g) (95% CI) measured by the 50c and the cortisol EIA of females and males in the control, diseased and injured groups are shown in Table 3. Mean, minimum and maximum FCM values for koalas of different conditions stratified by sex are presented in Appendix A, Table A5.

FCM values measured with the 50c EIA were significantly higher for the males in the control (*p* < 0.001) and diseased (*p* = 0.0210) animals, but did not differ between injured females and males (*p* = 0.2725). FCM values measured with the cortisol EIA were not significantly different between sexes for the three conditions, control (*p* = 0.2997), diseased (*p* = 0.9833) and injured koalas (*p* = 0.2042). Thus, in contrast to 50c EIA, cortisol EIA was not able to detect the difference in FCM values between males and females for control and diseased animals.

The results of the multivariate analysis are presented in Appendix B, Table A7. Although FCM values measured with 50c EIA were higher for males than females (*p* < 0.001) and higher in diseased and injured koalas compared to controls (*p* < 0.001), a significant interaction was also found between the conditions and the sex of the animals (*p* = 0.0307). Thus, the IRR of FCM values measured with 50c EIA were significantly lower for injured males relative to injured females when compared to the controls. Similarly, IRR of FCM values measured with 50c EIA were marginally lower for diseased males relative to diseased females when compared to the controls (*p* = 0.074).

In contrast, in the multivariate analysis for cortisol EIA, the interaction between the conditions and the sex of the animals could not be detected (*p* = 0.4221).

## 4. Discussion

This is the first long-term study conducted on hospitalised koalas comparing two different EIAs and using hospitalised and external controls. This is also the first study on rehabilitated koalas that considers the response of FCM values to the medication administered during rehabilitation.

Stress is associated with higher plasma cortisol levels, reflected in the faeces with an increased excretion of cortisol metabolites [20,32,41]. Hence, the aim here was to determine if diseases and injuries, as well as hospitalisation, would increase FCM values in wild koalas. Our hypothesis was that diseased and injured animals taken to hospital would show increased FCM values in the first scat collection, compared to the control. This hypothesis was based on the knowledge of the lag time between a stressful event and its reflection in FCM values, which is at least 10 h [32]. Hence, the faeces of diseased and injured koalas collected on day 1 would have shown altered values due to the prolonged pain that the koalas may have experienced prior to being admitted to hospital.

Our previous studies were conducted on koalas in wildlife parks [32,33]. They established that the 50c EIA is the most suitable assay for the detection of the koala-specific FCMs and determined the inability of a cortisol EIA to detect changes during the breeding season and to differentiate between sexes in captive koalas. They also established the stability of FCMs measured by the two assays [34]. These studies were in preparation for the current and future studies on wild koalas to establish levels of stress caused by anthropogenic factors [18,42], which may increase the susceptibility to diseases due to the inhibited immune response [11].

Some studies on stress in wildlife and domestic animals use cortisol EIAs to detect FCMs [28,43,44,45,46] during a variety of situations including hospitalisation and rehabilitation, albeit not validated in koalas [47]. Here, the use of the 50c EIA and cortisol EIA was justified to determine their suitability in free-living wild koalas and in those potentially stressed due to diseases, injuries and rehabilitation, to ensure that our previous studies on the detection of FCMs could be verified.

### 4.1. FCM Values for the Control Koalas from AZ and ML

The FCM values of both EIAs did not differ significantly between the AZ and the ML controls. However, the values of the two measured by the 50c EIA were more closely related. These values were also compared with our previous baseline study and it was found that the current values are equivalent or within the baseline values for both EIAs, but greater similarity is seen especially with the values detected by the 50c EIA (50c EIA: min = 2.2; max = 131.1; median = 16.5; cortisol EIA: min = 0.3; max = 33.9; median = 5.9) [33]. Many studies have established baseline values [48] of stress hormones before analysing the effect of climate change and other anthropogenic stressors on wildlife [41,49,50]. Previous, and current, results give us confidence that our baseline values can be used in this and future studies to detect stress in koalas. However, they can only be compared when exactly the same method (extraction and EIA) is used [28].

In contrast to our previous study [33], which determined a significant difference between FCM values during the breeding and non-breeding season detected only by the 50c EIA, here, this difference was not detected by either of the two EIAs. The difference in seasonal values in our previous study may have been due to the reproductive age of those koalas, causing seasonal fluctuations of FCMs, and by the consistent fortnightly collection of faecal pellets obtained by the same cohort of animals throughout the year. In this real-life study in wild koalas, the spread of age was unknown, however, juvenile koalas were part of the control cohort (e.g., Locket and Scratchy), which reduced the likelihood of detecting FCM fluctuations during the breeding season. Additionally, the number of koalas varied between the breeding and the non-breeding season and only one sample was analysed from each animal.

### 4.2. Longitudinal and 2-Day Scat Collection with and without GCs Treatment

A strong artificial increase in FCM values measured by the cortisol EIA was directly associated with the therapeutic systemic administration of prednisolone (Redipred) in koalas whose scats were collected during the therapy. These results can be explained by the antibody of the cortisol EIA cross-reacting with cortisone, prednisolone and prednisone [41,51,52] (the latter is an inactive metabolite formed from prednisolone [53]). Cross-reactions with these GCs also occur with the corticosterone assay (CJM006) [54]. The cortisol EIA used in this study (described by Palme and Möstl [27]), is not the same used by the authors above. Nevertheless, previous work in sheep [52] has shown cross-reaction with dexamethasone, hence, our findings, despite being unexpected, are not surprising (especially as prednisolone is more similar to cortisol than dexamethasone). While cortisol assays are used successfully for detecting stress in many species, their interaction with synthetic GCs can lead to incorrect interpretations and assumptions when the aim is to establish stress levels of rehabilitated/hospitalised animals if they are treated with GCs. The interpretation of this study could have been highly compromised if the longitudinal study of koalas treated with GCs had not been included in the research design. In fact, if only the 2-day sampling had been used to detect stress during hospitalisation, the assumption could have been that, at second collection time, the animals were highly stressed. In conclusion, it is a disadvantage that the cortisol EIA cross-reacts with synthetic GCs (metabolites), because it will bias FCM concentrations in treated koalas, thus disqualifying this EIA for evaluating naturally occurring stressors.

The GC treatment did not affect the values measured by the 50c EIA. This tetrahydrocorticosterone EIA has 20.7% cross-reactivity with tetrahydrocortisol (THF), the main FCM in koalas [32]. Moreover, the longitudinal study of koalas that were not treated with GCs shows that the increased values measured by the 50c and cortisol EIAs were related to stress caused by treatment and interventions during hospitalisation. Additionally, the cortisol EIA was unreliable in detecting stress in some koalas and was negatively correlated in some cases (Appendix A, Table A2). In fact, despite the high correlation between the FCM values of the two EIAs for the samples collected on day 1 in the three conditions, the correlation coefficients between 50c and cortisol EIA during the longitudinal study were low.

High endogenous GC levels trigger a negative feedback that decreases the secretion of the ACTH, consequently decreasing the release of endogenous GCs [5]. This regulation also occurs after the administration of synthetic GCs (e.g., during dexamethasone suppression test) in animal species and is also reflected in low FCM values (e.g., roe deer, wild subterranean rodents and ruminants [55,56,57,58]). However, negative feedback was not observed in some bird species (e.g., chickens [59]) and seasonal variations after the test have been observed in some horses and ponies [60,61]. Lack of dexamethasone suppression has also been observed in chukar, highly stressed by translocation [62], and in humans with high incidence of stress [63]. Here, the 50c EIA did not detect any negative feedback after the administration of synthetic GCs. With this study, we are not able to explain the lack of negative feedback in this group of koalas after the administration of synthetic GCs. However, it can be suggested that this may be either due to the low therapeutical dose (0.5 mg/Kg), the effect of stress caused by pain and hospitalisation, or the absence of negative feedback in this species. The latter two would have serious implications on the health of these animals if the negative feedback was absent in animals exposed to stressful events. In fact, the continuous release of endogenous GCs would affect the immune response [64] and increase the susceptibility of koalas to diseases, limiting conservation efforts [65,66]. It is suggested that further studies need to be undertaken to establish the physiological impact of synthetic GCs and, indeed, stress on the function of the HPA in koalas.

### 4.3. Impact of Disease and Injuries on FCM Values

As expected, on day 1, high FCM values were found in diseased and injured koalas. There is an abundance of literature on stress, including anthropogenic stress, causing illness and disease in wildlife, though there are no articles on the effect of disease on stress in wildlife. However, critical and acute illness, generally associated with serious inflammations and infections (bronchitis, meningitis, sepsis, etc.), had a strong impact on stress in children, causing a significant and sustained increase in plasma cortisol, which was, however, not significant in those with non-critical illnesses [67]. Hence, this increase in stress may be caused by the pain associated with infection and inflammation.

In fact, injuries are well known to cause pain and increase stress in humans and animals (domestic and wildlife). Pain in horses, resulting from colic, has shown to dramatically increase FCM values [26], and in giraffes, the increase in FCMs was proportional to the type of injury and hence the level of pain [25]. Elevated FCM values were also demonstrated in elephant bulls that had endured stress associated with prolonged pain [30]. Here, diseased and injured koalas had higher FCM values than the control when the animals were admitted to hospital. Moreover, koalas with more complications had higher FCM values, suggesting that levels of pain are reflected in the stress response. As stress impacts the immune system and, hence, the overall wellbeing of koalas with an impact on their conservation [18], it is important to diagnose stress and consider it in the treatment process during rehabilitation and before their release back into the wild to avoid disease recurrence.

### 4.4. Impact of Disease and Injuries on FCM Values between Sexes

The 50c EIA detected that FCM values of males were significantly higher than those of females (except for the injured), but no difference between sexes could be detected with cortisol EIA. Furthermore, through the interaction term between condition and sex in the multivariate analysis, it was identified that 50c EIA values were proportionally higher in females than males for individual conditions. This indicates that females, relative to males, respond to stressful situations derived from disease and injury with proportionally larger elevation of FCM values when measured with 50c EIA. In contrast, when measuring stress levels with cortisol EIA, a difference between sex and the interaction between the sexes and the three conditions could not be detected (despite the detection of higher FCM values in the diseased and injured groups compared to healthy koalas). Thus, the cortisol EIA is less suited to detecting subtle differences between physiological changes due to pain.

Therefore, in koalas, the cortisol EIA had a lesser discriminatory power than the 50c EIA, which reiterates what was already demonstrated in our previous work [33]. However, in other species, cortisol EIAs were able to differentiate FCM values between female and male tigers [68] and other carnivores, albeit with inconsistencies [51]. This confirms that the metabolism of cortisol is species-specific and may differ between sexes [3,69], and that not all EIAs are suited for use with every species [46]. A reliable EIA, which does not cross-react with synthetic GCs, is needed to evaluate stress in koalas. Otherwise, as we found for cortisol EIA, results would be confounded in situations where a treatment with such exogenous GC is necessary. As tetrahydrocortisol has been identified as the main FCM in koalas, the 50c EIA, due to its valuable characteristic of cross-reacting with tetrahydrocortisol rather than prednisone, is suitable to detect stress in this species without interference from administered, synthetic GCs. Hence, so far, the 50c EIA has proved to be the most suitable and reliable EIA for the detection of stress in koalas.

### 4.5. Limitations

This study was designed to assess the stress caused by injuries and disease and the effect of hospitalisation on wild koalas. While this study successfully achieved its aim, there were some limitations worth mentioning here.

Due to the real-life nature of this study, some elements could not be standardised. As these were wild koalas, their age was not known and, hence, the FCM values between age groups could not be established. However, our previous investigation on captive koalas of known age [33], showed that there was no significant difference in FCM values between age groups. Nevertheless, those were healthy animals, so further studies should analyse, where possible, differences between FCM values and age groups of compromised animals.

Koalas were admitted to hospital at different times, but this would not alter the FCM values of day 1 samples as these reflected stress events occurred many hours earlier and further samples in the hospital were collected freshly defecated each morning. Nevertheless, our previous study [33] showed differences in FCM values between morning and evening (only detected by the 50c EIA). However, the values shown by the stressed animals in this study were much higher than the percentage difference between morning and evening medians in the captive cohort of the previous project. Hence, these differences may be disregarded, considering the high values of stressed animals observed in this study.

## 5. Conclusions

This study has clearly demonstrated that pain causes an increase in stress in koalas, which is known to impact the immune system. Stressed koalas have an increased likelihood of being further affected by illnesses, hindering their natural recovery and wellbeing.

While we have established a link between stress and diseases and injuries, anthropogenic activities, some also causing climate change, are also considered stressors increasingly impacting on the survival of koalas. Using a species-specific EIA for measuring FCM values, as an indicator of stress, is of utmost importance.

Here, it has been demonstrated that stress can be reliably measured with the 50c EIA. It has been confirmed that the baseline FCM values measured in our previous paper can be used to assess stress in koalas. It has been shown that, before admission to hospital, the diseased and injured koalas were stressed.

More importantly, this study has clearly demonstrated the unsuitability of the cortisol EIA to determine stress in most rehabilitated animals and in those treated with synthetic GCs, as the values detected reflect administered exogenous GCs rather than the endogenous cortisol metabolites. Hence, future studies on stress of rehabilitated animals medicated with GCs need to take into consideration the cross-reaction of the EIA with any exogenous GCs. These findings will be applied to a wider study on wild koalas where the relationship between health and stress will be further investigated.

## Figures and Tables

**Table 1:** Koalas used for the study: 146 from the Australia Zoo Wildlife Hospital (code: AZ) and 88 from a study conducted by Endeavour Veterinary Ecology at The Mill (code: ML).

AZ				ML
Diseased ^1^	Injured ^2^	Diseased + Injured	Control ^3^	External control ^4^
76 (42F + 34M)	30 (11F + 19M)	4 (2F + 2M)	36 (19F + 17M)	88 (44F + 44M)
Non-breeding season N = 74 Breeding season N = 72				Non-breeding season N = 76; Breeding season N = 12

^1^ Mostly affected by urogenital and ocular chlamydiosis; ^2^ Injured by dogs and cars; ^3^ Taken to AZ by concerned citizens and considered as the AZ control; ^4^ Considered external control; F = Female; M = Male. Non-breeding season = February–August; Breeding season = September–January.

**Table 2:** Summary statistics for FCM (faecal cortisol metabolite) values (ng/g) measured by 50c and cortisol EIAs (enzyme immunoassays) for first and second scat collection (between the 10th and 15th day) for the control (N = 7), diseased (N = 32) and injured (N = 13).

Condition	N	Sampling Point	Mean Days between Samplings	Median 50c EIA (ng/g) (95% CI)	50c EIA *p*-Value *	Median Cortisol EIA (ng/g) (95% CI)	Cortisol EIA *p*-Value *
Control	7	1		19.7 (13.2, 32.3)		9.7 (3.1, 25.4)	
		2	12.2	12.4 (8.8, 30.2)	0.125	3.3 (2.2, 11.6)	0.4531
Diseased	32	1		33.5 (26.0, 50.2)		9.8 (6.3, 14.3)	
		2	13.2	36.2 (25.0, 52.3)	0.4869	9.3 (5.7, 19.4)	0.8601
Injured	13	1		34.2 (26.2, 38.8)		9.8 (5.2, 15.5)	
		2	12.9	30.0 (20.9, 42.2)	0.5811	8.8 (4.5, 11.0)	0.0225

* Wilcoxon sign rank test for matched samples.

**Table 3:** Median FCM values (ng/g) (95% CI) measured by 50c and cortisol EIAs (enzyme immunoassays) for females and males in the control, diseased and injured group.

	50c EIA (ng/g) (95% CI)		Cortisol EIA (ng/g) (95% CI)	
	Females	Males	Females	Males
Control (Females N = 63 Males N = 61)	14.4 (12.1, 17.2)	29.4 (23.3, 36.5)	6.4 (5.5, 7.2)	6.8 (6.0, 8.1)
Diseased (Females N = 42 Males N = 34)	29.2 (20.6, 43.1)	44.7 (33.4, 57.4)	11.4 (9.3, 17.8)	11.0 (8.9, 16.2)
Injured (Females N = 11 Males N = 19)	29.5 (16.6, 52.6)	35.7 (32.7, 40.7)	11.8 (6.0, 33.6)	6.6 (5.1, 12.5)

## Data Availability

Raw data are available from the corresponding author upon reasonable request.

## Appendix A

**Table A1:** Summary statistics for FCM (faecal cortisol metabolite) levels (ng/g) measured by 50c and cortisol EIAs (enzyme immunoassays) for control koalas in each location (AZ = Australia Zoo Wildlife Hospital; ML = The Mill).

Location		50C EIA (ng/g)	Cortisol EIA (ng/g)
AZ (N = 36)	Min. Max. Mean	5.5 72.6 26.9	2.2 40.1 10.6
ML (N = 88)	Min. Max. Mean	1.9 85.6 23.2	2.2 19.5 7.5

**Table A2:** Longitudinal study graphs showing the effect of hospital treatment on FCM (faecal cortisol metabolite) values measured by 50c (continuous line) and cortisol (dashed line) EIAs.

Graphs Reflecting FCM Values	Notes and Correlations
(a)	Locket (female): control The relatively high values on day 1 are likely related to the hospitalisation the afternoon before day 1. Correlation coefficient between FCM values measured by the cortisol and 50c EIA: Pearson’s r = −0.0385 (*p* = 0.9347)
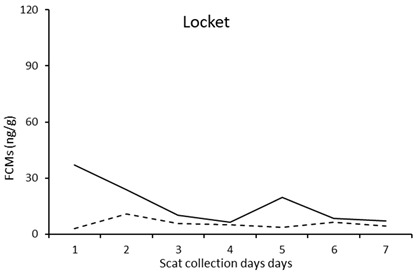
(b)	Scratchy (male; orphan): control The high values on day 1 are likely related to the hospitalisation the day before day 1 after she was found alone on the side of a road. Correlation coefficient between FCM values measured by the cortisol and 50c EIA: Pearson’s r = −0.18442 (*p* = 0.5818)
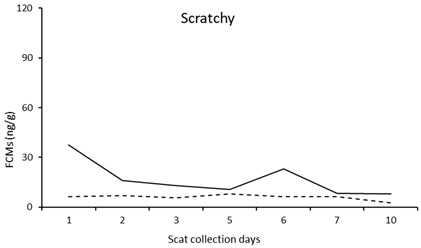
(c)	Cloyna (male): diseased (cystitis, anaemia and kidney disease) He had extreme values of FCMs at admission due to illness. The spike on day 2 is likely related to the hospitalisation on day 1. He was euthanised after the last faeces collection. Correlation coefficient between FCM values measured by the cortisol and 50c EIA: Pearson’s r = −0.0019 (*p* = 0.9962)
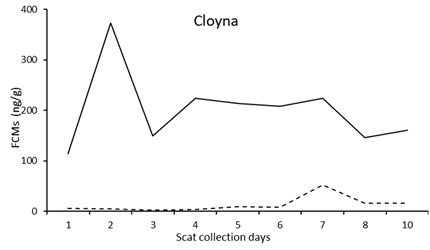
(d)	Cyril (female): diseased (*Chlamydia;* LAMP = 13.37 min) The spike on day 2 is likely related to the hospitalisation on day 1. Correlation coefficient between FCM values measured by the cortisol and 50c EIA: Pearson’s r = 0.1000 (*p* = 0.8311)
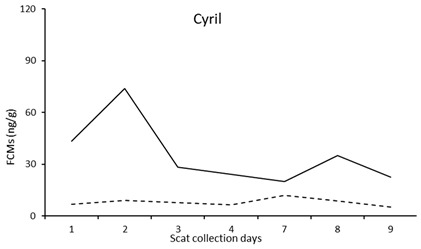
(e)	Larissa (female): diseased (*Chlamydia;* LAMP = 3.56 min) The high values from day 7 are likely related to the start of the injection treatment. Correlation coefficient between FCM values measured by the cortisol and 50c EIA: Pearson’s r = −0.6866 (*p* = 0.0600)
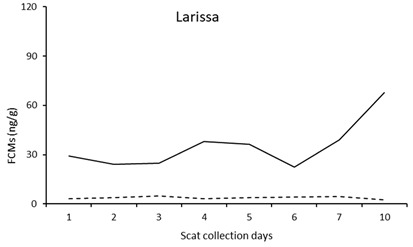
(f)	Molly (female): diseased (*Chlamydia;* LAMP = 15.15 min) Treatment started on day 4. Correlation coefficient between FCM values measured by the cortisol and 50c EIA: Pearson’s r = 0.3144 (*p* = 0.6064)
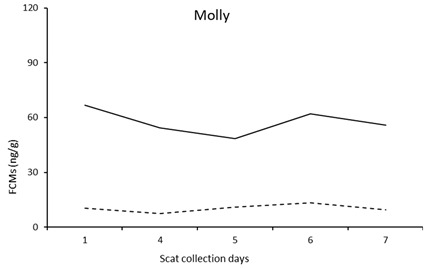
(g)	Golf (male): diseased (*Chlamydia*; LAMP = 14.4 min) The spikes on days 2 and 6 are likely related to the procedure for general anaesthetic carried out on days 1 and 5. Correlation coefficient between FCM values measured by the cortisol and 50c EIA: Pearson’s r = 0.4136 (*p* = 0.2348)
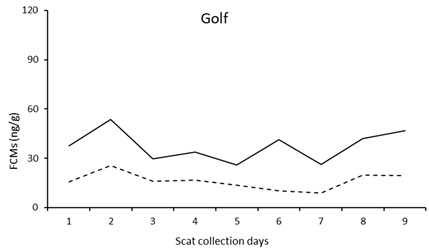
(h)	Clementine (female): diseased (*Chlamydia:* LAMP = 8.51 min) No specific data were recorded but she was ultimately euthanised. Correlation coefficient between FCM values measured by the cortisol and 50c EIA: Pearson’s r = 0.8439 (*p* = 0.0084)
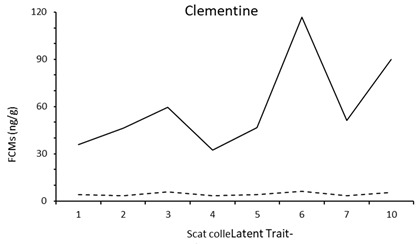
(j)	Rarsta (male): diseased (kidney infection) He underwent injection treatment. Rarsta was euthanised 3 days after the last scat collection. Correlation coefficient between FCM values measured by the cortisol and 50c EIA: Pearson’s r= 0.4579 (*p* = 0.2539)
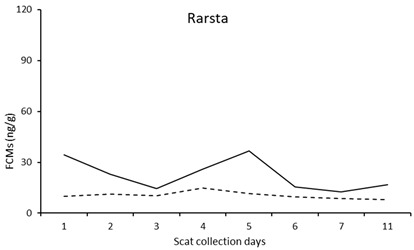
(k)	Blake (male): diseased (chlamydial conjunctivitis) The spike on day 4 is likely related to day 3 injection. Correlation coefficient between FCM values measured by the cortisol and 50c EIA: Pearson’s r= 0.8270 (*p* = 0.0113)
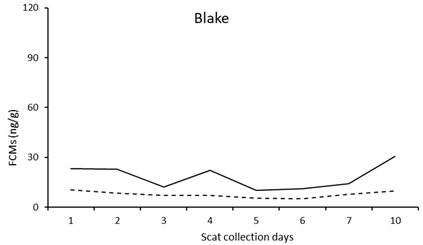
(l)	Spring (female): diseased (chlamydial conjunctivitis) She was recorded to be highly stressed in her enclosure on day 3. No notes were available to explain the increase on day 7. Correlation coefficient between FCM values measured by the cortisol and 50c EIA: Pearson’s r = 0.4835 (*p* = 0.2249)
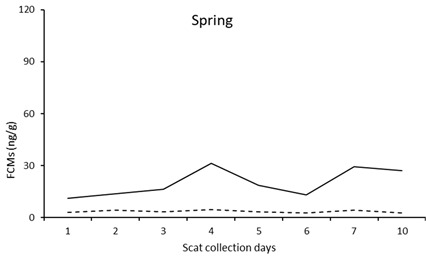
(m)	Wayne-o (male): injured (hit by car) The spike on day 2 is likely related to the hospitalisation on day 1. Procedure for general anaesthetic was carried out on day 5. Correlation coefficient between FCM values measured by the cortisol and 50c EIA: Pearson’s r = −0.1518 (*p* = 0.7197)
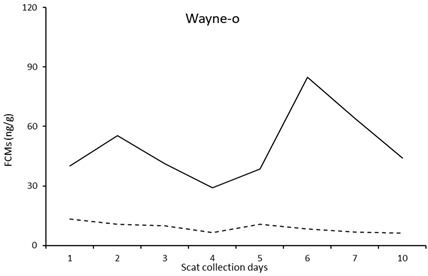
(n)	Firecracker (female): injured (hit by a car) The spike on day 2 is likely related to the procedure for general anaesthetic carried out on day 1. Correlation coefficient between FCM values measured by the cortisol and 50c EIA: Pearson’s r = 0.4041 (*p* = 0.2808)
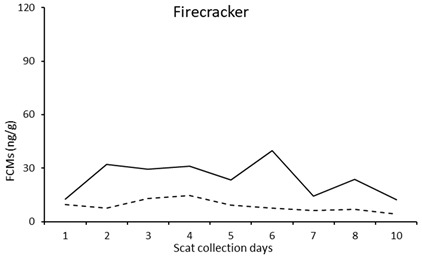
(o)	Milko (male): injured (hit by a car) Injection treatment started on day 1. Procedure for general anaesthetic was carried out on days 2 and 4. Correlation coefficient between FCM values measured by the cortisol and 50c EIA: Pearson’s r = 0.1905 (*p* = 0.6235)
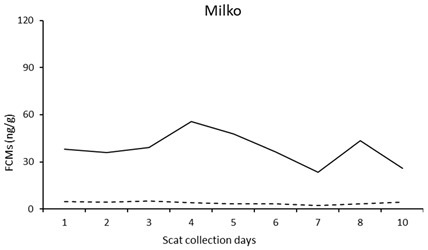
(p)	Farrel (male): injured (abscess on the face and peritonitis) He was administered an injection of antibiotic on day one and carprofen, starting on day 2 for 5 days. Correlation coefficient between FCM values measured by the cortisol and 50c EIA: Pearson’s r = 0.2169 (*p* = 0.6403)
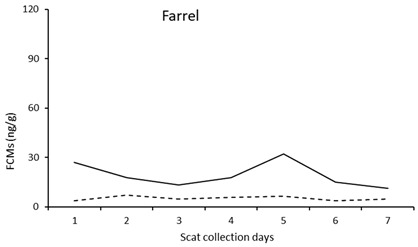
(q)	Luca (male): injured The high values on day 1 are likely related to the hospitalisation in the afternoon before day 1. No data were recorded that may explain the spike on day 4. The higher values from day 7 are likely related to the procedure for general anaesthetic carried out on day 7. Correlation coefficient between FCM values measured by the cortisol and 50c EIA: Pearson’s r = 0.3763 (*p* = 0.3582)
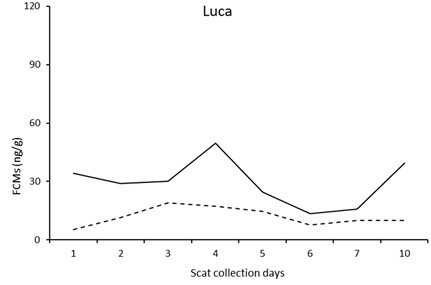
(r)	Noah (male): injured (dog attack) The spike on day 2 is likely related to the hospitalisation in the afternoon before day 1. Noah was kept in the ICU. No other information is available that could explain the spike on day 7. Correlation coefficient between FCM values measured by the cortisol and 50c EIA: Pearson’s r = 0.6886 (*p* = 0.0699)
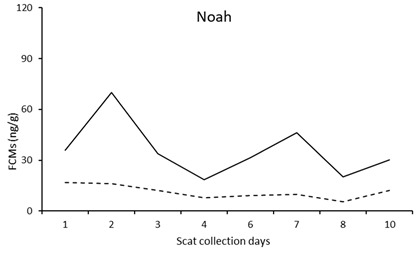

**Table A3:** Koalas whose second scat sample was collected after the administration of GCs (glucocorticoids) was terminated.

Name	GCs Last Day Administration	Second Scat Collection
Paddy	13 June	15 June
Noodle	21 October	25 October
Florence	11 March	16 March
Nita	31 August	2 September
Deville	2 October	24 November

**Table A4:** Summary statistics for FCM (faecal cortisol metabolite) values (ng/g) measured by 50c and cortisol EIAs (enzyme immunoassays) for the control, diseased and injured groups.

Group		50C EIA (ng/g)	Cortisol EIA (ng/g)
Control (N = 124)	Min. Max. Mean	1.9 85.6 24.3	2.2 40.1 8.4
Diseased (N = 76)	Min. Max. Mean	7.4 135.7 41.2	1.9 86.6 18.2
Injured (N = 30)	Min. Max. Mean	7.9 371.4 47.9	2.8 90.5 15.2

**Table A5:** Summary statistics for FCM (faecal cortisol metabolite) values (ng/g) measured by 50c and cortisol EIAs (enzyme immunoassays) for females and males in the control, diseased and injured groups.

		50c EIA (ng/g)		Cortisol EIA (ng/g)	
		Females	Males	Females	Males
Control (Females N = 63 Males N = 61)	Min. Max. Mean	1.9 52.4 16.8	4.5 85.6 32.1	2.2 31.4 7.6	2.8 40.1 9.2
Diseased (Females N = 42 Males N = 34)	Min. Max. Mean	7.4 83.5 34.8	8.8 135.7 49.1	1.9 77.1 17.7	2.6 86.6 19.0
Injured (Females N = 11 Males N = 19)	Min. Max. Mean	7.9 371.4 62.0	10.6 120.0 39.7	2.9 58.2 19.0	2.8 90.5 13.1

## Appendix B

**Table A6:** FCM (faecal cortisol metabolites) values measured by the 50c and the cortisol EIAs (enzyme immunoassays) for the control koalas during breeding and non-breeding seasons on the first collection day.

	50c EIA (ng/g) (95% CI)		Cortisol EIA (ng/g) (95% CI)	
	Breeding (N = 90)	Non-Breeding (N = 34)	Breeding (N = 90)	Non-Breeding (N = 34)
Median (95% CI)	20.6 (18.2, 27.3)	17.2 (13.7, 22.4)	6.4 (5.7, 7.2)	6.8 (5.1, 10.6)
Mean	25.4	21.5	8.1	9.1
Min/max	1.9/85.6	4.5/72.6	2.2/40.1	2.2/35.5

**Table A7:** Multivariate analysis of FCMs (faecal cortisol metabolites) for females and males and interaction with the three conditions (control, diseased and injured) measured with 50c and cortisol EIA (enzyme immunoassays).

50c EIA	IRR 95%CI (Low, High)	*p*-Value	Wald Test *p*-Value
**Sex**			
Female	1		
Male	1.9 (1.5, 2.3)	<0.001	
**Condition**			
Control	1		<0.001
Diseased	2.0 (1.6, 2.6)	<0.001	
Injured	3.6 (1.4, 9.6)	0.008	
**Sex # Condition**			
Male#Control vs. Female#Control	1		0.0307
Male#Diseased vs. Female#Diseased	0.7 (0.5, 1)	0.074	
Male#Injured vs. Female#Injured	0.3 (0.1, 0.9)	0.033	
**Cortisol EIA**	**IRR 95%CI (low, high)**	***p*** **-value**	**Wald test** ***p*-value**
**Sex**			
Female	1		
Male	1.2 (0.9, 1.5)	0.135	
**Condition**			
Control	1		<0.001
Diseased	2.3 (1.6, 3.1)	0	
Injured	2.5 (1.4, 4.2)	0.001	
**Sex # Condition**			
Male#Control vs. Female#Control			0.4221
Male#Diseased vs. Female#Diseased	0.8 (0.5, 1,4)	0.636	
Male#Injured vs. Female#Injured	0.5 (0.2, 1.3)	0.2	
